# Introducing the Stem Cell ASCL2 Reporter STAR into Intestinal Organoids

**DOI:** 10.1016/j.xpro.2020.100126

**Published:** 2020-10-08

**Authors:** Maria C. Heinz, Koen C. Oost, Hugo J.G. Snippert

**Affiliations:** 1 Molecular Cancer Research, Center for Molecular Medicine, University Medical Center Utrecht, University Utrecht, Utrecht, The Netherlands; 2 Oncode Institute, Utrecht, The Netherlands

## Abstract

Patient-derived organoids maintain functional and phenotypic characteristics of the original tissue such as cell-type diversity. Here, we provide protocols on how to label intestinal (cancer) stem cells by integrating the stem cell ASCL2 reporter (STAR) into human and mouse genomes via two different strategies: (1) lentiviral transduction or (2) transposon-based integration. Organoid technology, in combination with the user-friendly nature of STAR, will facilitate basic research in human and mouse adult stem cell biology.

For complete details on the use and execution of this protocol, please refer to Oost et al. (2018).

## Highlights

• Choose the optimal STAR plasmid suited for your research
• Tips and tricks on how to prepare organoids for STAR integration
• Diverse protocols for STAR integration: lentiviral and transposon-based approaches

## Graphical Abstract

**Figure:**
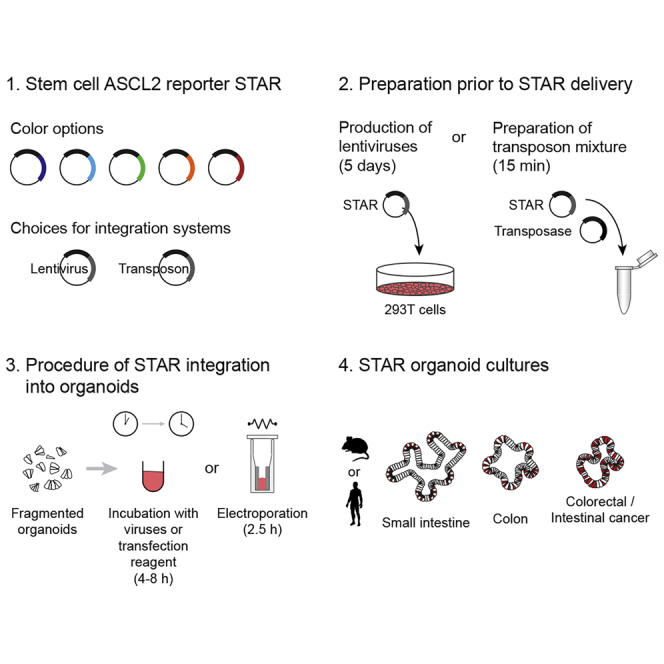

## Teaser

Patient-derived organoids maintain functional and phenotypic characteristics of the original tissue such as cell-type diversity. Here, we provide protocols on how to label intestinal (cancer) stem cells by integrating the stem cell ASCL2 reporter (STAR) into the human and mouse genome via two different strategies: (1) lentiviral transduction or (2) a transposon-based system. Organoid technology, in combination with the user-friendly nature of STAR, will facilitate basic research in human and mouse adult stem cell biology.

## Before You Begin

**Figure 1:**
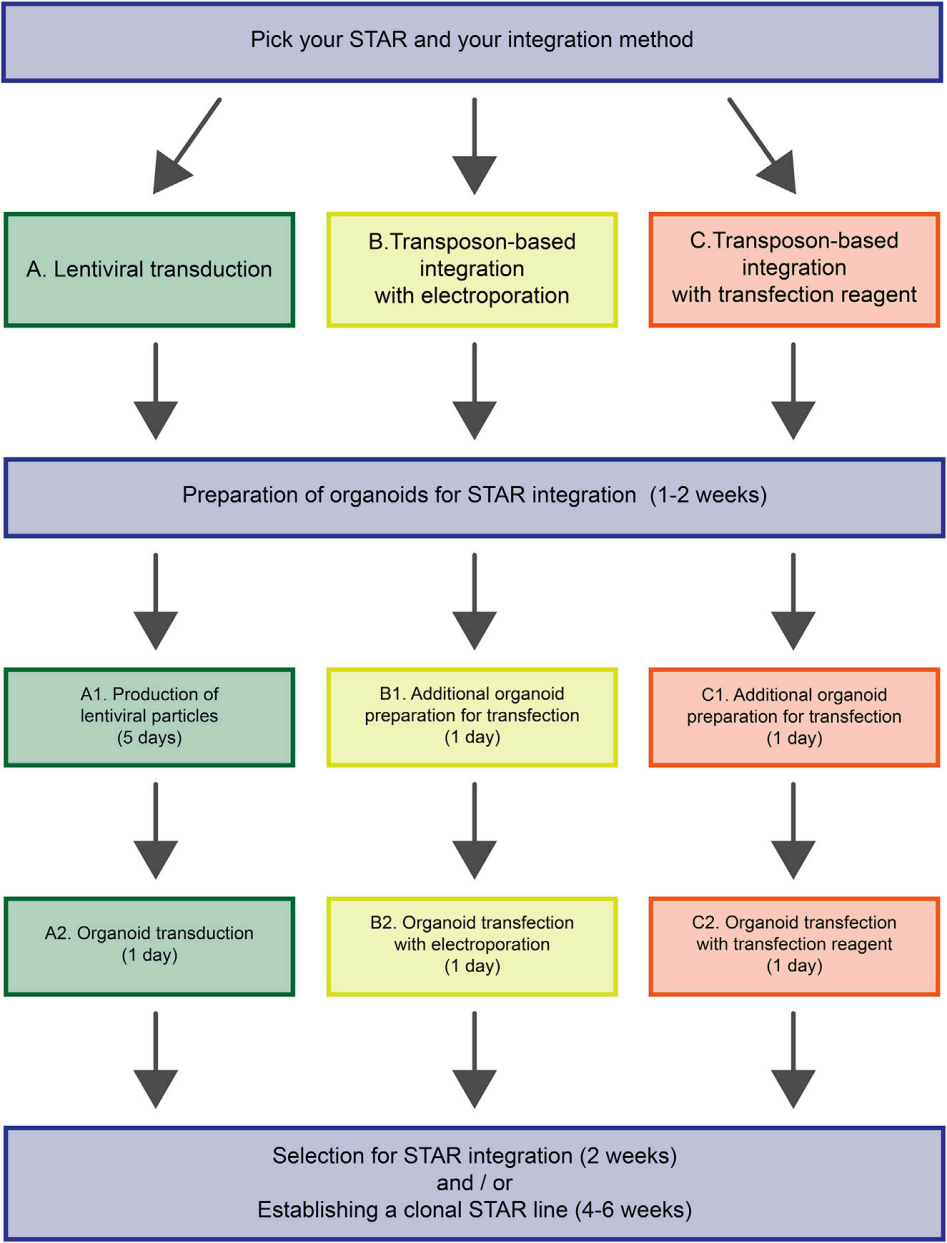
Workflow for Integrating STAR into Organoids Flow chart indicating the key steps with time estimates for the different integration methods provided: lentiviral transduction (protocol A, green), transposon-based integration with electroporation (protocol B, yellow), and transposon-based integration with a transfection reagent (protocol C, red). Protocol steps required for all integration methods are highlighted in blue.

A schematic workflow of the protocol options is provided in Figure 1.

### Choosing Your STAR Minigene

**Figure 2:**
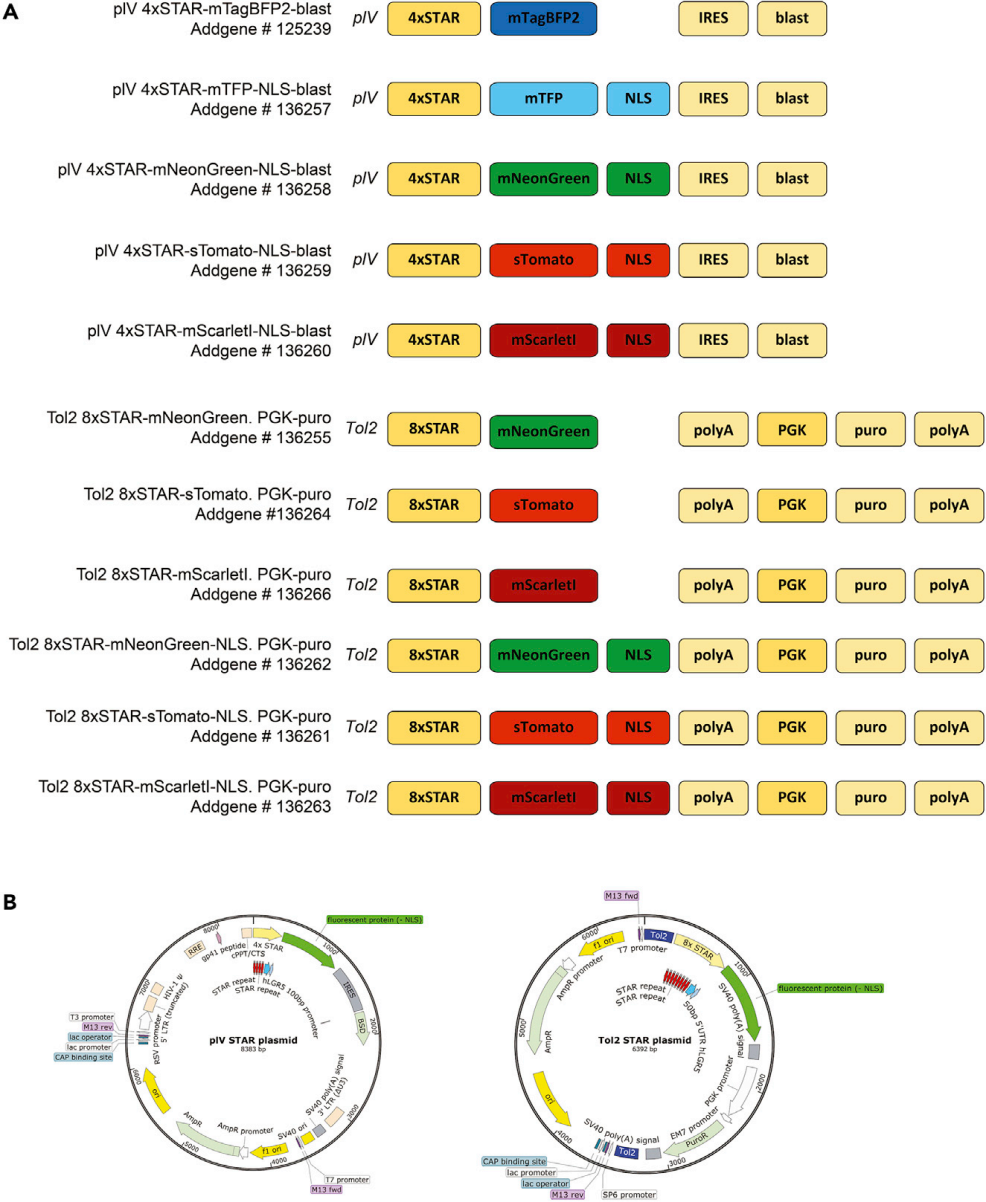
The STAR Plasmid Selection (A) Schematic overview of available STAR reporter designs suitable for lentiviral (plV) or Tol2 transposon-based integration (Tol2). STAR contains either 4 or 8 repeats of the ASCL2 binding motif (4xSTAR and 8xSTAR, respectively). Further features: fluorescent protein, nuclear localization signal (NLS), selection cassette conferring resistance to puromycin (puro) or blasticidin (blast), internal ribosome entry site (IRES), polyadenylation signal (polyA), and independent, ubiquitously active PGK promoter. Addgene names and numbers of all plasmids are listed. See also Table 1. (B) Schematic overview of plasmid vector maps (STAR and backbone) for lentiviral (left) and transposon-based integration (right).

The minigene STAR faithfully marks mouse and human intestinal stem cells (Oost et al., 2018) by reporting the transcriptional activity of ASCL2, the master regulator of intestinal cell fate (Schuijers et al., 2015). STAR can be used to identify, track, and study intestinal stem cells using fluorescent labels, which vary in their spectral properties. We have generated STAR constructs with varying fluorescent proteins, subcellular localizations, selection cassettes, and integration strategies (Figure 2 and Table 1). The choice of the optimal STAR variant depends on the specific research question and related requirements should be met.

**Table 1:** STAR Selection at a Glance

Resource	Integration Method	STAR Color	Selection Antibiotic	Addgene Number
pLV 4xSTAR-mTagBFP2-blast	pLV	TagBFP2	blasticidin	125239
pLV 4xSTAR-mTFP-NLS-blast	pLV	mTFP	blasticidin	136257
pLV 4xSTAR-mNeonGreen-NLS-blast	pLV	mNeonGreen	blasticidin	136258
pLV 4xSTAR-sTomato-NLS-blast	pLV	sTomato	blasticidin	136259
pLV 4xSTAR-mScarletI-NLS-blast	pLV	mScarletI	blasticidin	136260
Tol2 8xSTAR-mNeonGreen. PGK-puro	Tol2	mNeonGreen	puromycin	136255
Tol2 8xSTAR-sTomato. PGK-puro	Tol2	sTomato	puromycin	136264
Tol2 8xSTAR-mScarletI. PGK-puro	Tol2	mScarletI	puromycin	136266
Tol2 8xSTAR-mNeonGreen-NLS. PGK-puro	Tol2	mNeonGreen	puromycin	136262
Tol2 8xSTAR-sTomato-NLS. PGK-puro	Tol2	sTomato	puromycin	136261
Tol2 8xSTAR-mScarletI-NLS. PGK-puro	Tol2	mScarletI	puromycin	136263

List of all available STAR plasmids generated by the Snippert lab and provided to Addgene. Plasmids are either suitable for lentiviral-based (pLV) or transposon-based (Tol2) integration. The fluorescent color reporting STAR activity and selection antibiotics are listed for each plasmid. For a graphical representation of the STAR plasmids see Figure 2.

The robustness of a fluorescent reporter is determined by multiple parameters such as expression levels, fluorophore properties, and potentially genomic integration site. In this section, we discuss features to be considered to obtain optimal signal from the STAR minigene.

1. Applicability of the STAR minigene:
a. STAR can be used to fluorescently label cells with transcriptional activity of ASCL2 in human and murine cells. To our knowledge, it has not been thoroughly tested for other species yet.
b. In particular, STAR can be used to faithfully mark small intestinal (SI) and colonic stem cells in both mouse and human organoids, while its expression pattern overlaps with Lgr5 expression as demonstrated for mouse SI organoids (Oost et al., 2018).
c. STAR can also be used to mark stem-like cells in colorectal cancer. Recent evidence suggests that not all patient-derived colorectal cancer cell lines express ASCL2 (Jubb et al., 2006; Ziskin et al., 2013), which constitutes a natural but hitherto not encountered limit.
d. STAR might be suitable to reflect the transcriptional activity of ASCL2 in organoid systems beyond the intestine of men and mice. However, this has not been tested yet.
e. When STAR is expected to target a non-stem cell population, a ubiquitously expressed selection cassette is preferable. For more details see section “Selection Procedure for STAR Organoids.”
f. As Wnt signaling drives ASCL2 expression (Van der Flier et al., 2007), supplementing the organoid medium with exogenous Wnt may lead to a high fraction of STAR^+^ cells with moderate differences in STAR levels. Cellular differentiation protocols can lead to a higher degree of cellular diversity by reducing the amount of stemness-promoting factors. Consequently, these media induce crypt-(like) budding structures and promote the difference between STAR-high and STAR-negative cells (Sato et al., 2011).
g. Studies previously performed using STAR organoids: STAR has been used to study the cellular composition of human colon and patient-derived CRC organoids. Colony formation assays have demonstrated the stem cell properties of single STAR^+^ cells in human colon organoids. Additionally, STAR has been used to profile tumor subpopulations transcriptomically and to visualize cancer stem cells in xenograft studies (Oost et al., 2018).
2. Comparison of STAR to endogenous labeling of stem cells:
a. STAR is a very versatile tool, allowing to generate a large number of reporter lines simultaneously in a relatively short time frame with a high success rate (thus maintaining polyclonality).
b. As STAR is reporting the transcriptional activity of ASCL2, the brightness of the fluorescent protein is not limited by minimum expression level of an endogenous stem cell marker gene. This makes STAR superior for time-lapse microscopy studies and studies on cell fate conversions (differentiation or plasticity).
c. Visualization of intestinal stem cells in human organoids has been performed, for instance by generating a CRISPR/Cas9-mediated knockin into the LGR5 locus (Fujii et al., 2018). By endogenously tagging the LGR5 protein with a fluorescent protein, both the subcellular localization and interaction partners of LGR5 can be reliably studied.
d. Genetic lineage tracing studies provide easy readout for assessing a cell’s self-renewal and multipotency potential. Using LoxP-STOP-LoxP-based reporter systems and a knockin of CreER into the LGR5 locus, this has been successfully performed in human colonic organoids (Shimokawa et al., 2017; Sugimoto et al., 2017).
3. Cellular localization of the fluorescent protein:
We have generated STAR plasmids with and without a nuclear localization signal (NLS) fused to the fluorescent protein. Their key features are listed below:
a. Nuclear localization
i. Excellent for quantifications and cell tracking
ii. In combination with a bright fluorophore, more difficult to distinguish expressions levels with basic microscopy as the fluorescent proteins are concentrated in a smaller volume
b. Uniform localization
i. Displays cellular morphology (e.g., stem cells are slender cells in mouse small intestinal organoids)
ii. Excellent for co-expression studies with cytoplasmic (stem cell) markers
4. Features of fluorescent proteins
a. To capture ASCL2 dynamics with high accuracy during time-lapse microscopy, the ideal fluorophore would have to have a high extinction coefficient and quantum yield (brightness), high photostability (limited bleaching), a short maturation time, and a high protein turnover. Most of these criteria are met by either mNeonGreen or sTomato, however, depending on the research question a different fluorophore might be more suitable.
b. When planning to use the STAR lines for in vivo experiments, using a fluorescent protein with an established antibody provides highest flexibility in terms of downstream processing and allows, for instance, for paraffin-embedding or Edu chemistry and subsequent retrieval of the STAR signal.
5. Microscope setup
Generally speaking, the different fluorophores used in this study are suitable for many microscopy techniques as the proteins can be excited with either of the following laser options: 405 nm, 458 nm, 488 nm, or 561 nm. If, however, STAR shall be combined with multiple different colors which leads to limited detection windows, it might be preferable to choose bright fluorescent proteins which can be most optimally excited with the available laser equipment.

### Choosing Your Integration Strategy

We provide STAR plasmids that can be integrated into the genome of organoids either by lentiviral transduction or by transposase-based approaches.

Protocol A: Lentiviral integration
Recommended if high expression levels are desired and for organoids that are very difficult to transfect
Protocol B: Transposon-based integration with electroporation
Recommended if medium expression levels are desired and for organoids which are difficult to transfect. Expected to lead to fewer integrations than lentiviral approach. Transfection might also be suitable for induced pluripotent stem cells or embryonic stem cell approaches.
Protocol C: Transposon-based integration with transfection reagent
Suitable for organoids that are readily transfectable

All three integration strategies are suitable for the generation of organoid lines with the following features: species: mouse or human, organ: small intestine or colon, genome: WT or cancerous. In our experience, organoid lines with a WT genome might be more difficult to transfect with a transfection reagent (approach C). Hence, in situations transposon-based integration with electroporation might be superior (approach B). An overview of the experimental workflows is provided in Figure 1.

### Preparation of Organoid Cultures

**Timing: 1–2 weeks**

**Figure 3:**
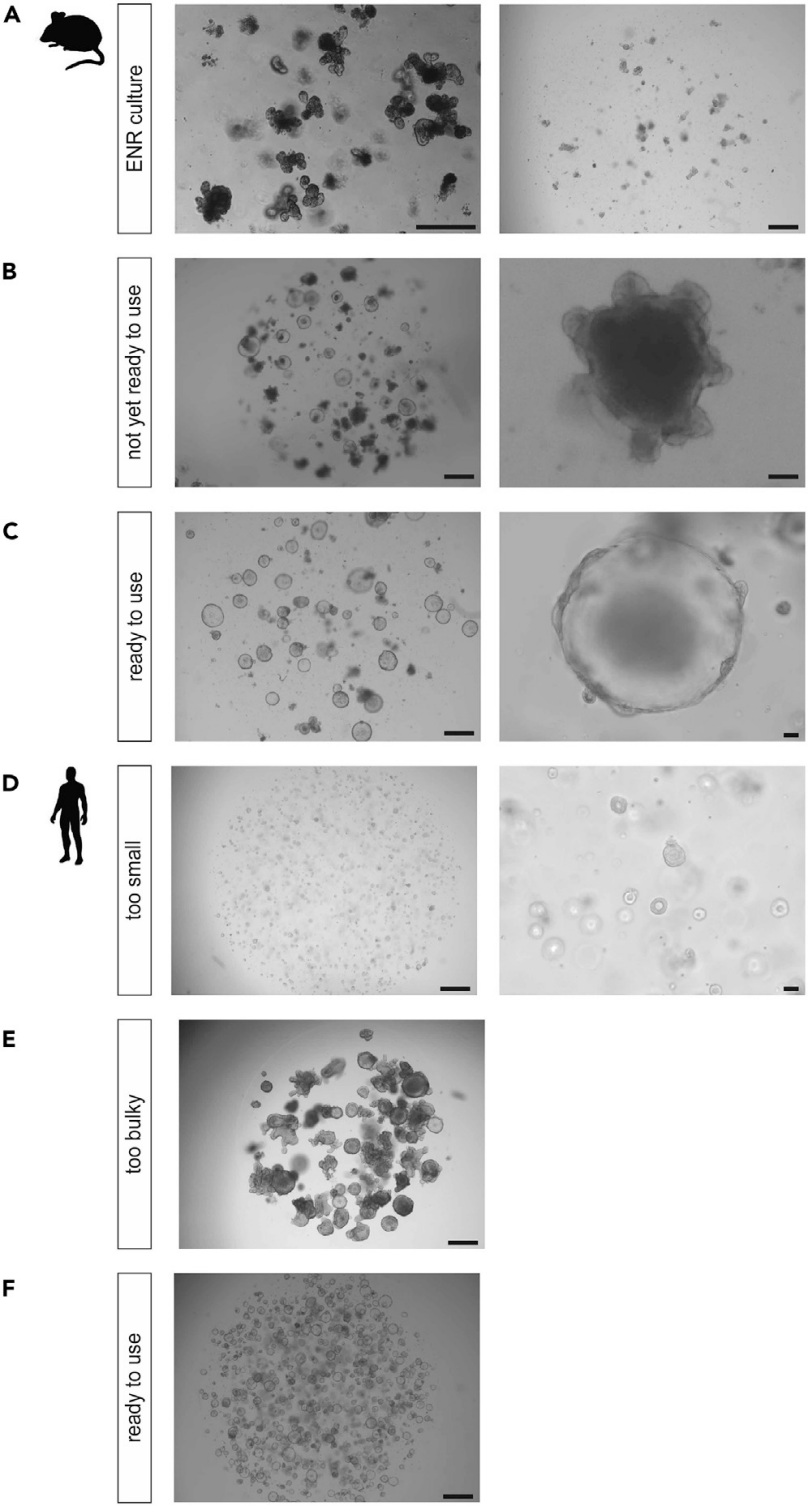
Preparation of Organoids Prior to STAR Integration (A–C) Mouse small intestinal organoids. (A) Organoids in ENR medium. Mature culture (left) and culture directly after passaging by mechanical fragmentation (right). (B) Changing from ENR to WENR medium yields highly proliferative cyst-like structures over time (right panel = close up (scale bar 50 μm)). (C) Organoid culture consistently depicting cyst-like phenotypes that is ready to use for STAR integration. (right panel = close up (scale bar, 50 μm)). (D–F) Human colonic organoids. (D) Organoid culture is not dense enough / organoids are too small to reach sufficient cell numbers. (right panel = close up (scale bar, 50 μm)). (E) Organoids are too bulky / too much differentiated. (F) Organoids are in a highly proliferative state and ready for STAR integration. Scale bars, 500 μm unless indicated otherwise.

To maximize the efficiency of generating stable STAR organoids, the organoids should be in a healthy, **proliferative state** prior to starting any transduction/transfection protocol (Figure 3).

**CRITICAL:** (for mouse SI organoids) We recommend reverting mouse SI organoids from their regular crypt-like morphology (ENR culture medium) into a highly proliferative state (WENR culture). To get to this state, mouse SI organoids should be cultured in WENR medium (see section “Materials and Equipment / General Organoid Media”) after passaging by mechanically shearing off the crypt units. When most of the organoids demonstrates a cystic morphology (usually after 5–10 days), organoids can be used for STAR integration (Figure 3C).

For complete details on the use and execution of this protocol paragraph, please refer to Koo et al. and Andersson-Rolf et al. for the preparation of mouse SI organoids for transfection or transduction (Andersson-Rolf et al., 2014; Koo et al., 2012).

***Note:*** To obtain **enough starting material**, it is advisable to expand the organoid culture for 1–2 weeks prior to STAR integration. For electroporation-based transfection, the success rate increases significantly if 500,000—1 million cells are being electroporated. This corresponds to organoids growing in approx. 200–400 μL Matrigel for 7–10 days after the last passage.

**CRITICAL:** (for transfection-based approaches) As serum hampers the transfection efficiency, organoids should be cultured with **serum-free medium** for the transfection-based approaches (procedures B and C), starting one day in advance. This implies leaving out Penicillin/Streptomycin and replacing the Wnt CM with 10 μM CHIR99021 (see Step-By-Step Method Details).

**Table:** 

Preparation Step	Lentiviruses	Transfection with Electroporation	Transfection with Transfection Reagent
Reverting mouse SI organoids to a proliferative state using WENR medium	Recommended	critical	critical
Expanding the culture to get a high cell number (500,000 or more)	Obsolete	critical	recommended
Culture in serum-free media prior to STAR integration	Obsolete	critical	critical

## Key Resources Table

**Table:** 

REAGENT or RESOURCE	SOURCE	IDENTIFIER
**Chemicals, Peptides, and Recombinant Proteins**		

Matrigel	Corning	Cat# 356231
Dispase II	Life Technologies	Cat# 17105041
Trypsin EDTA	Sigma-Aldrich	Cat# 25200056
Trypsin inhibitor	Sigma-Aldrich	Cat# T9003
Opti-MEM	Life Technologies	Cat# 31985054
Advanced DMEM/F-12	Invitrogen	Cat# 12634-028
GlutaMAX	Invitrogen	Cat# 35050-038
HEPES	Invitrogen	Cat# 15630-056
Penicillin/Streptomycin	Lonza	Cat# DE17-602E
EGF (Recombinant Human EGF)	PeproTech	Cat# AF-100-15
B27 supplement	Invitrogen	Cat# 17504001
N-Acetyl-L-Cysteine	Sigma-Aldrich	Cat# A9165
Nicotinamide	Sigma-Aldrich	Cat# N0636
A83-10	Tocris	Cat# 2939/10
SB202190	Gentaur	Cat# A1632
Y-27632	Gentaur	Cat# A3008
Primocin	Invivogen	Cat# ant-pm-2
Puromycin	Sigma-Aldrich	Cat# P7255
Blasticidin	Bio Connect	Cat# ant-bl-1
CHIR99021 (Protocols B and C)	Bio-Techne	Cat# 4423/10
Dulbecco’s Modified Eagle’s Medium (DMEM) – high glucose (Protocol A)	Sigma-Aldrich	Cat# D6429
Fetal bovine serum (FBS, Protocol A)	Fisher Scientific	Cat# 10309433
Polybrene/Hexadimethrine bromide (Protocol A)	Merck	Cat# 107689

**Experimental Models: Cell Lines**		

HA-RSpondin1-Fc 293T cell line to make R-Spondin CM	In-house production	N/A
HEK293-mNoggin-Fc cell line to make Noggin CM	In-house production	N/A
L-Wnt3A cell line to make Wnt CM	In-house production	N/A
HEK293T cell line (Protocol A)	N/A	N/A

**Recombinant DNA**		

Envelope plasmid for lentiviral production pHDM-G (Protocol A)	PlasmID by DF/HCC DNA Resource Core at Harvard Medical School	PlasmID clone ID EvNO00061607
Helper plasmid for lentiviral production pHDM-Hgpm2 (Protocol A)	PlasmID by DF/HCC DNA Resource Core at Harvard Medical School	PlasmID clone ID EvNO00061606
Helper plasmid for lentiviral production pRC-CMV-rev1b (Protocol A)	PlasmID by DF/HCC DNA Resource Core at Harvard Medical School	PlasmID clone ID EvNO00061616
CMV-Tol2 transposase (Protocols B and C)	Addgene	Addgene 158774

**Other**		

24-well tissue culture plates	Magazijn	Cat# 3524
Cell culture dishes, 100 mm × 20 mm	Greiner	Cat# 664160
X-tremeGENE 9 DNA transfection reagent (Protocols A and C)	Life Technologies	Cat# 6365779001
Sterile 50 mL concentric luer-lock syringe (Protocol A)	BD Plastipak	Cat# 300865
Sterile 0.45 μm syringe filter (Protocol A)	Satorius	Cat# 16555-K
Ultracentrifuges tubes, e.g., Thinwall Polypropylene Tube, 25 × 89 mm (Protocol A)	Beckman Coulter	Cat# 326823
BTX electroporation solution (Protocol B)	Fisher Scientific	Cat# 15417350
Electroporation cuvette Plus, BTX, 2 mm gap (Protocol B)	Fisher Scientific	Cat# 15447270

## Materials and Equipment

### The STAR Selection

A selection of STAR plasmids for either lentiviral or Tol2 transposon-based integration has been generated and is depicted in Figure 2 and Table 1. The plasmid sequences at base-pair resolution are available on Addgene.

### General Organoid Media

The organoid culture media were established by Sato et al. (Sato et al., 2009, 2011).

**Table:** 

Ingredient	Mouse SI organoids, ENR culture	Mouse SI organoids, WENR culture	Human SI/colon WT culture
**Advanced DMEM/F12 Supplemented with GlutaMAX, HEPES, Pen/Strep**			

Wnt 3A CM		50%	50%
R-Spondin CM	5%	20%	20%
Noggin CM	10%	10%	10%
EGF (Recombinant Human EGF)	50 ng/mL	50 ng/mL	50 ng/mL
B27 supplement	2%	2%	2%
N-Acetyl-L-Cysteine	1.25 mM	1.25 mM	1.25 mM
Nicotinamide		10 μM	10 μM
A83-01			500 nM
SB202190			3 μM

***Note:*** In case of organoid lines with pathway-activating mutations, certain culture ingredients can be left out (Drost et al., 2015).

Troubleshoot 9: Access to conditioned media

### Additional Organoid Media Required for Lentiviral Transduction (Protocol A)

**Transduction medium:** Adjust standard organoid medium as follows

• Add 10 μM Y-27632
• Add 8 μg/mL polybrene

***Optional:* Freezing medium for lentiviruses:** Adjust standard organoid medium as follows

• If standard organoid medium contains Wnt CM, replace it by 10 μM CHIR99021
• Add 10 μM Y-27632
• Add 8 μg/mL polybrene

### Additional Organoid Media Required for Transposon-Based Integration (Protocols B and C)

**CRITICAL:** Transfection medium should be serum-free. Due to the dissociation procedure, the following adaptations are recommended:

**Transfection medium:** Adjust standard organoid medium as follows

• Use Advanced DMEM/F12 supplemented with GlutaMAX and HEPES (but without Pen/Strep)
• If standard organoid medium contains Wnt CM, replace it by 10 μM CHIR99021
• Add 10 μM Y-27632

### Additional Equipment Required for Lentiviral Integration (Protocol A)

• Ultracentrifuge, e.g., Beckman Coulter with SW32Ti rotor

***Alternatives****:* If you do not have access to an ultracentrifuge, you can also concentrate the virus using the Lenti-X ™ concentrator according to the manufacturer’s instructions (Takara, Cat. No.: 631231) with which we have limited but good experience.

### Additional Equipment Required for Electroporation-Based Integration (Protocol B)

• Electroporation machine, e.g., Nepa Gene NEPA21

## Step-By-Step Method Details

### Integration of STAR into the Genome

The protocols below describe the integration of STAR via (A) lentiviral transduction, (B) transposase-based integration with electroporation, and (C) transposase-based integration using a transfection reagent.

#### Protocol A: Lentiviral Transduction

**Timing: 5 days**

For complete details on the use and execution of this part of the protocol, please refer to Van Lidth de Jeude et al. for the organoid transduction protocol with lentiviruses (Van Lidth de Jeude et al., 2015).

#### Protocol A1: Production of Lentiviral Particles

This protocol describes the production of lentiviral particles using VSV-G envelopes. All lentiviral STAR plasmids listed in Table 1 are suitable for 3rd generation lentivirus making with VSV-G envelopes and the packaging vectors gag, pol, and rev. Transduced stem cells will generate STAR organoids.

**CRITICAL:** Protocol steps involving lentiviral particles must be performed in a BSL-2 lab (indicated below). This involves careful attention to BSL-2 regulations on sample handling and disposal of plasticware and media.

Day 1:

**Timing: 30 min**

1. Split HEK293T cells to 25%–30% confluency in T75 flask or 100 mm petri dish and plate in DMEM supplemented with 10% fetal bovine serum (DMEM/FBS).

***Note:*** Each plate at 80% confluency can be split over 4 plates.

Day 2:

**Timing: 25 min**

2. Prepare transfection solution as indicated in the table below.

Troubleshoot 2: No access to indicated lentiviral packaging plasmids or transfection reagent

3. Mix transfection solution thoroughly, and incubate at 20°C for 15 min.

**CRITICAL:** Do not incubate the transfection mixture for more than 30 min.

**Table:** 

Reagent	For 1 Dish	For 2 Dishes	For 4 Dishes
Opti-MEM	100 μL	200 μL	400 μL
DNA STAR reporter stock concentration at 1 μg/μL	5 μL	10 μL	20 μL
DNA pMDLg/pRRE stock concentration at 1 μg/μL	1 μL	2 μL	4 μL
DNA pRSV-Rev stock concentration at 1 μg/μL	1 μL	2 μL	4 μL
DNA pMD2.G stock concentration at 1 μg/μL	2 μL	4 μL	8 μL
X-tremeGENE 9	18 μL	36 μL	72 μL

**CRITICAL:** The following step must be performed in a BSL-2 lab.

4. Drip 127 μL DNA transfection solution onto HEK293T cells growing in DMEM/FBS and store them in a humidified cell culture incubator at 37°C.

Day 3:

**Timing: 5 min**

**CRITICAL:** The following step must be performed in a BSL-2 lab.

5. Carefully aspirate medium from HEK293T culture plates and add 8 mL DMEM/FBS to each plate.

***Note:*** This culture media volume allows to pool the virus suspension of up to 4 plates into one bucket for ultracentrifugation on Day 5.

Day 5:

**Timing: 3.5 h, including 2.5 h centrifugation**

**CRITICAL:** The following steps must be performed in a BSL-2 lab.

6. Before you begin:
a. Pre-cool ultracentrifuge to 4°C
b. Prepare transduction medium as described in section “Additional Organoid Media Required for Lentiviral Transduction (Protocol A)”
c. ***Optional:*** Prepare freezing medium for lentiviruses as described in section “Additional Organoid Media Required for Lentiviral Transduction (Protocol A)”
7. ***Optional:*** If dead cells are floating in the culture medium, collect supernatant in 15 mL flask and centrifuge for 5 min at 500 × *g*. Continue with supernatant.
8. Collect the culture medium from the plates and push it through a 0.45 μm filter using a large 60 mL syringe.
9. Load ultracentrifuge buckets with tubes and pipet virus suspension into the tube.

***Note:*** Culture media of up to 4 plates can be combined into one ultracentrifugation bucket.

10. Carefully equal balance opposite buckets (lids included) by topping up with DMEM/FBS. Afterwards, carefully cap buckets with corresponding lids.
11. Centrifuge for 2.5 h at 170,000 × *g* at 4°C.
12. In the meantime, prepare N∗550 μL transduction medium with N being the total number of flasks or plates in which virus has been produced.
13. Transfer ultracentrifuge buckets very carefully into a laminar flow hood, remembering the orientation of the tube inside the centrifuge.
14. Open bucket containing ultracentrifuge tube and decant medium carefully in such orientation that the opaque brown pellet is on the upper side of the tube. Take a micropipette and remove leftover medium, while taking care not to agitate the pellet.
15. Resuspend the virus pellet in N∗500 μL of organoid transduction medium.
16. Use 250 μL virus suspension (N/2) to transduce organoids grown in 50–100 μL Matrigel (see protocol A2). Residual virus suspension can be aliquoted in single-use fractions (250 μL each) and frozen at −80°C.

**Pause Point:** The lentivirus can be frozen at −80°C and transduction can be done on a different day with a minor loss in efficiency.

***Note:*** In our experience, 2-year old virus stocks are suitable for high efficiency transductions (minor loss compared to fresh virus), when previously stored as single-use aliquots at −80°C.

#### Protocol A2: Lentiviral Transduction of Organoids

**Timing: 3.5–6.5 h, including 1 h centrifugation and 1–4 h incubation**

**CRITICAL:** Protocol steps involving lentiviral particles must be performed in a BSL-2 lab (indicated below). This involves careful attention to BSL-2 regulations on sample handling and disposal of plasticware and media.

17. Before you begin:
a. Pre-heat water bath to 37°C
b. Pre-cool centrifuge to 4°C
18. Harvest organoids with ice-cold medium and transfer into a 15 mL tube.

***Note:*** For one transduction, 50–100 μL of Matrigel containing approx. 5,000–20,000 organoids which were passaged 7–10 days ago should suffice.

19. Top up to 10 mL with ice-cold Advanced DMEM and spin at 4°C, 300 × *g* for 5 min.

***Note:*** Ice-cold medium will dissolve the Matrigel.

20. Discard supernatant.
21. ***Optional:*** If residual Matrigel is visible (not a clean pellet), repeat steps 19 and 20.
22. Discard supernatant and resuspend pellet in 500 μL trypsin. Incubate for about 3 min in a 37°C water bath until organoid have dissociated into single cells or small fragments.

***Note:*** (Non-cancerous) Organoids might have reduced outgrowth potential when trypsinized to single cells.

23. Inactivate trypsin by adding 500 μL trypsin inhibitor (1:1 ratio with trypsin), top up to 10 mL with Advanced DMEM, and spin at 4°C, 500 × *g* for 5 min.
24. Discard the supernatant and resuspend pellet in 20 μL transduction medium.

**CRITICAL:** The following steps have to be performed in a BSL-2 lab.

25. Add 250 μL virus suspension to the organoids and spin at RT, 75 × *g* for 1 h.
26. Incubate samples in a 37°C incubator for 1–4 h with a loosened lid.

***Note:*** Prolonged incubation of up to 4 h may increase the transduction rate significantly.

27. In the meantime, thaw Matrigel on ice and keep on ice after thawing.
28. Spin samples at RT, 500 × *g* for 5 min.
29. Take off supernatant and resuspend cell pellet in 100–200 μL Matrigel. Plate in 10 μL droplets onto a pre-warmed 24-well plate with 4–5 droplets per well.
30. Incubate plate upside-down in the incubator for 10–20 min until the Matrigel has solidified.

***Note:*** Turning the plate upside-down prevents organoids from sinking to the bottom of the culture plate and attaching to the plastic.

31. Add 500 μL organoid medium supplemented with 10 μM Y-27632 to each well.

Start with selection procedure, 3 days after transduction (see section “Selection Procedure for STAR Organoids”).

Troubleshoot 1: STAR integration by lentiviruses

Troubleshoot 3: Cell death upon transduction

#### Protocol B: Transposon-Based Integration with Electroporation

**Timing: 3 days**

For complete details on the use and execution of this part of the protocol, please refer to Fujii et al. for the organoid electroporation protocol (Fujii et al., 2015).

32. Pre-heat water bath to 37°C
33. Pre-cool centrifuge to 4°C
34. Thaw Matrigel on ice
35. Prepare electroporation mixture as follows: For each electroporation condition mix 100 μL BTX electroporation solution + 10 μL STAR reporter DNA (stock concentration 1 μg/mL)+ 5 μL miniTol2 transposase(stock concentration 1 μg/mL).
36. Label collection tubes for after electroporation.
37. Prepare 100× dispase: Dilute 0.05 g Dispase II in 500 μL Advanced DMEM/F12 and mix well by pipetting and vortexing until completely dissolved.
38. Prepare 1× dispase (inside a flow cabinet): Add 100 μL of 100× dispase stock to 9.9 mL Advanced DMEM/F12 and mix by vortexing briefly.

***Note:*** Dispase stocks (100× and 1×) can be stored at 4°C but dispase is most active during the first days after preparation.

39. Prepare transfection medium as described in section “Additional Organoid Media Required for Transposon-Based Integration (Protocols B and C)”

#### Protocol B1: Preparation of Organoids for Transfection

Day 1:

**Timing: 5 min**

40. Replace organoid medium with transfection medium while using 500 μL transfection medium for each well of a 24-well plate.

***Note:*** 200–400 μL Matrigel containing organoids grown for 7–10 days after the last passage should suffice for one electroporation.

Day 2:

**Timing: 1–1.5 h**

41. Add 1.25% v/v DMSO to the transfection medium.
42. Keep organoids in the incubator for 2–4 h.
43. Take off medium, collect organoid droplets using 1× dispase, and transfer into a 15 mL tube.
44. Incubate in 37°C water bath for 3–10 min until Matrigel is properly dissolved.

***Alternatives* to steps 43 and 4**4***:*** Harvest organoids with ice-cold medium and transfer into 15 mL tube. This approach might require additional washing/centrifugation steps as indicated in step 46.

45. Top up to 10 mL with cold Advanced DMEM/F12 and spin at 4°C, 300 × *g* for 5 min.
46. If a clean organoid pellet is visible, discard the supernatant. Otherwise, carefully take off the clear supernatant on top of the Matrigel cloud (approx. 8 mL of supernatant). Then, repeat steps 45 and 46.
47. Resuspend pellet in 1 mL of trypsin. Incubate for about 3 min in a 37°C water bath until organoid have dissociated into small fragments.

***Note:*** Dissociation to small fragments, rather than to single cells, will increase the survival and outgrowth potential upon electroporation.

48. Inactivate trypsin by adding 1 mL of trypsin inhibitor (1:1 ratio with trypsin), top up to 10 mL with Advanced DMEM/F12, and spin at 4°C, 500 × *g* for 5 min.
49. ***Optional:*** Discard the supernatant, resuspend the pellet in 1 mL Advanced DMEM/F12, and roughly estimate the cell number by counting. 500,000–1 million cells are recommended for one electroporation. Afterwards, pellet cells again by spinning at 4°C, 500 × *g* for 5 min.
50. Discard the supernatant and put tube on ice.

#### Protocol B2: Transfection of Organoids Using Electroporation

**Timing: 45 min**

51. Set up the electroporator. During the subsequent steps, avoid prolonged incubation of the cells in electroporation buffer.
52. Resuspend cell pellet in 115 μL electroporation mixture and transfer 100 μL into electroporation cuvette.
53. Cap the cuvette with its lid and place the electroporation cuvette in the cuvette holder of the electroporator.
54. Measure the impedance and adjust if value outside 30–55.

***Note:*** Increasing the volume in the cuvette will lower the impedance.

55. ***Optional:*** If impedance is larger than 30, add residual cell suspension to the cuvette to increase the number of cells which are being electroporated.
56. Electroporate cells with the settings indicated in the table below. Troubleshoot 4: Cell death upon electroporation
57. Add 400 μL of Opti-MEM supplemented with 10 μM Y-27632 to the electroporation cuvette.
58. Use the plastic pipette provided with the cuvette to transfer the cells from the cuvette into an Eppendorf tube and put on ice.
59. Rest cells for 10–20 min on ice.
60. Spin tubes at 4°C, 500 × *g* for 5 min.
61. Take off supernatant.
62. Resuspend pellet in approx. 200 μL Matrigel and plate cells at high density in 10 μL droplets onto a pre-warmed 24-well plate with 4–5 droplets per well.

***Note:*** A significant fraction of cells will not survive this harsh procedure. Hence, cells can be plated more densely than usual for maintenance of the organoid line.

63. Incubate the plate upside-down in the incubator for 10 min until the Matrigel has solidified.
64. Add 500 μL transfection medium + 1.25% DMSO per well for at least 30–60 min.
65. Refresh each well with 500 μL transfection medium without DMSO.

**Table:** 

	Poring pulse	Transfer Pulse
Voltage	175 V	20 V
Pulse Length	5 ms	50 ms
Pulse interval	50 ms	50 ms
Number of pulses	2	5
Decay rate	10%	40%
Polarity	+	+/−

Day 3:

**Timing: 5 min**

66. Replace medium with 500 μL standard culture medium.

Start with selection procedure, 3–7 days after transfection (see section “Selection Procedure for STAR Organoids”).

Troubleshoot 1: Transposon-based STAR integration via electroporation

#### Protocol C: Transposon-Based Integration Using a Transfection Reagent

The protocol for organoid transfection with transfection reagents was inspired by a transfection protocol using liposomes of Schwank et al. (Schwank et al., 2013), while the key transfection step was adapted by the Snippert lab.

67. Pre-heat water bath to 37°C
68. Pre-cool centrifuge to 4°C
69. Prepare 100× dispase: Dilute 0.05 g Dispase II in 500 μL Advanced DMEM/F12 and mix well by pipetting and vortexing until completely dissolved.
70. Prepare 1× dispase (inside a flow cabinet): Add 100 μL of 100× dispase stock to 9.9 mL Advanced DMEM/F12 and mix by vortexing briefly.

***Note:*** Dispase stocks (100× and 1×) can be stored at 4°C but dispase is most activeduring the first days after preparation.

71. Prepare transfection medium as described in section “Materials and Equipment/Additional Organoid Media Required for Transposon-Based Integration (Protocols B and C) ”

#### Protocol C1: Preparation of Organoids for Transfection

**Timing: 3 days**

***Note:*** Protocol is written for the transfection of one well of a 24-well plate containing 50 μL Matrigel. Sufficient organoid amounts for successful transfection are between 4 and 8 wells of a 24-wells (approx. 200–400 μL Matrigel).

Day 1:

**Timing: 5 min**

72. Replace organoid medium with 500 μL transfection medium per well.

Day 2:

**Timing: 1–1.5 h**

73. Take off medium, collect organoid droplets using 1× dispase, and transfer into a 15 mL tube.
74. Incubate in 37°C water bath for 3–10 min until Matrigel is properly dissolved.

***Alternatives* to steps 73 and 74*:*** Harvest organoids with ice-cold medium and transfer into 15 mL tube. This approach might require additional washing/centrifugation steps as indicated in step 76.

75. Top up to 10 mL with Advanced DMEM and spin at 4°C, 300 × *g* for 5 min.
76. If a clean organoid pellet is visible, discard the supernatant. Otherwise, carefully take off the clear supernatant on top of the Matrigel cloud (approx. 8 mL of supernatant). Then, repeat steps 75 and 76.
77. Discard supernatant and resuspend pellet in 250 μL of trypsin. Incubate for about 3 min in a 37°C water bath until organoid have dissociated into single cells or small fragments.

***Note:*** (Non-cancerous) Organoids might have reduced outgrowth potential when trypsinized to single cells.

78. Inactivate trypsin by adding 250 μL of trypsin inhibitor (1:1 ratio with trypsin), top up to 10 mL with Advanced DMEM, and spin at 4°C, 500 × *g* for 5 min.
79. Take off supernatant and put cells on ice.

#### Protocol C2: Transfection of Organoids with a Transfection Reagent

**Timing: 6–7 h, including 5–6 h incubation**

80. Prepare transfection mixture as follows: For one well mix 50 μL Opti-MEM + 0.5 μL STAR reporter DNA at 1 μg/mL + 0.25 μL miniTol2 transposase at 1 μg/mL + 2.25 μL X-tremeGENE 9. Problem: No access to X-tremeGENE 9

**Recommended:** If more wells (N wells) are used for transfection, scale transfection mixture linearly.

81. Mix transfection mixture well by vortexing, spin the tube briefly to remove drops from the lid or the side of the tube, then incubate for 15 min at RT.

**CRITICAL:** Do not incubate the transfection mixture for more than 30 min.

82. Resuspend cell pellet in 225 μL transfection medium (previously prepared as described at the beginning of this protocol).
83. Add N∗53 μL transfection mixture to the cell suspension with N being the total number of wells the organoids were derived from and mix well by pipetting up and down.
84. Incubate for 5–6 h in the incubator with a loosened lid.
85. Toward the end of the incubation period, thaw Matrigel on ice and keep on ice after thawing.
86. Spin tubes at 4°C, 500 × *g* for 5 min.
87. Take off supernatant.
88. Resuspend pellet in Matrigel and plate cells in 10 μL droplets onto a pre-warmed 24-well plate with 4–5 droplets per well.
89. Incubate the plate upside-down in the incubator for 10 min until the Matrigel has solidified.
90. Add 500 μL transfection medium to each well.

Day3:

**Timing: 5 min**

91. Replace medium with 500 μL standard culture medium.

Start with selection procedure, 3 days after transfection (see section “Selection Procedure for STAR Organoids”).

Troubleshoot 1: Transposon-based STAR integration via transfection reagent

### Selection Procedure for STAR Organoids

**Timing: 3 days to 2 weeks**

**Figure 4:**
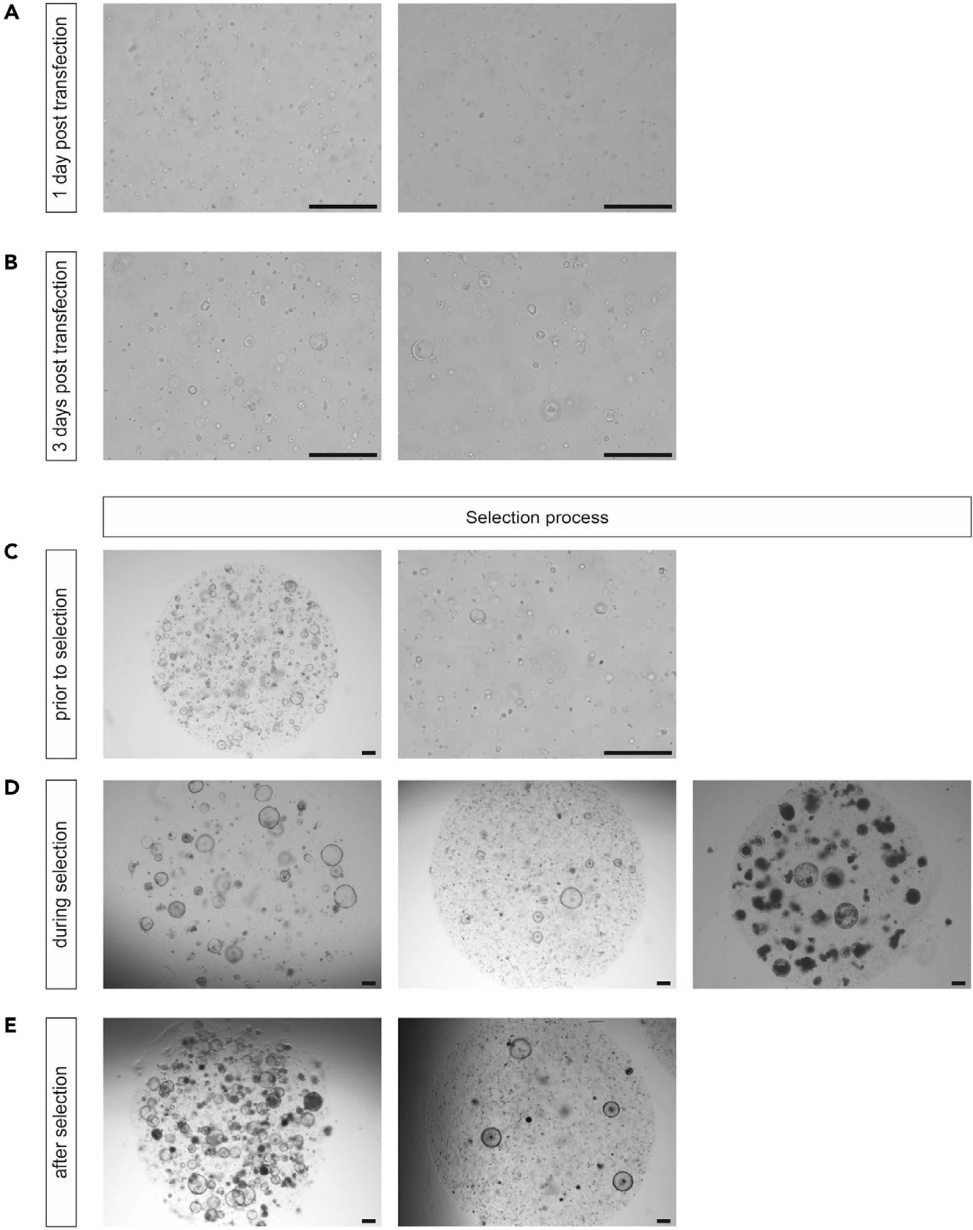
Organoids during STAR Integration (A–C) Electroporated organoid culture prior to selection. (A) Individual cells, one day after electroporation. (B) Small organoid structures at day 3. (C) Representative image of 4–7 day old viable organoids on which selection can be applied. (D) Organoid cultures during the selection process. Efficiency of integration may fluctuate per condition (high on the left, low in the middle). Late administration of selection antibiotics (e.g., day 7) results in dead organoid structures (right panel), rather than dead single cells. (E) Organoids having survived the selection procedure for high (left) and low (right) STAR integration efficiency. Scale bars, 250 μm.

Selecting for STAR organoids can be started as early as 3 days post integration. If viability is low (usually after electroporation), start selecting 7 days post integration to not further disturb the outgrowth process. Representative figures of organoids prior, during, and after selection are depicted in Figure 4. (The fraction of surviving organoids is representative for STAR integration by transfection. Lentiviral transduction rates usually are between 20%–80% in our hands.)

**Timing: 5 min**

92. Refresh medium and supplement with the appropriate selection antibiotic (puromycin at 1–4 μg/mL and blasticidin at 2–50 μg/mL).

***Optional:*** As the sensitivity to the drugs might vary across lines, a dose-response test on non-transduced/transfected organoids might be informative.

***Note:*** We usually use concentrations which kill off negative clones within 3 and 7 days for puromycin and blasticidin, respectively. In our experience, organoid lines with a WT genome can be selected for using drug concentrations at the lower end of the indicated spectrum, while cancerous organoids might require higher concentrations.

#### Selection in Case of a Ubiquitously Expressed Resistance Cassette

**Timing: 1–2 weeks**

93. Keep organoids on selection medium for at least 1–2 passages to ensure proper selection.

***Note:*** Organoids are most vulnerable at a single cell state which is why passaging organoids to single cells during the selection process is recommendable. Exceptions to this may be organoid lines with a poor single cell outgrowth efficiency.

94. ***Optional:*** FACS-purify STAR^+^ cells and grow them into a culture.

***Note:*** This procedure is suitable in particular if a polyclonal line shall be used for future experiments. (For more detail, see first note below.)

95. ***Optional:*** Pick individual organoids grown from a single cell state to establish a clonal culture [see section “Establishing a clonal STAR organoid line”].

#### Selection in Case of a STAR-Driven Resistance Cassette

**Timing: 1–2 weeks**

96. Keep organoids on selection medium for about 3–5 days to enrich for STAR^+^ cells.
97. Continue to culture organoids in the absence of the selection drug.
98. **Recommended:** FACS-purify STAR^+^ cells and grow them into a line.

***Note:*** This procedure is suitable to establish a polyclonal line and could be used for future experiments. (For more detail, see first note below.)

99. ***Optional:*** Pick individual organoids grown from a single cell state to establish a clonal culture [see section “Establishing a clonal STAR organoid line”].

***Note:*** We recommend to perform this step after FACS-purifying STAR^+^ cells (step 98) to ensure that reporter-positive clones are picked.

***Note:*** FACS purification of STAR^+^ cells is suitable for all organoid lines in which STAR^+^ cells constitute the stem cell population and therefore regenerate the organoid culture. It further ensures proper selection for cells having integrated the reporter which is particularly reassuring in cases of transient antibiotics selection. For designs in which a separate promoter drives the expression of a selection cassette, FACS purification of STAR^+^ cells rules out partial silencing of the reporter at the STAR sites.

**Figure 5:**
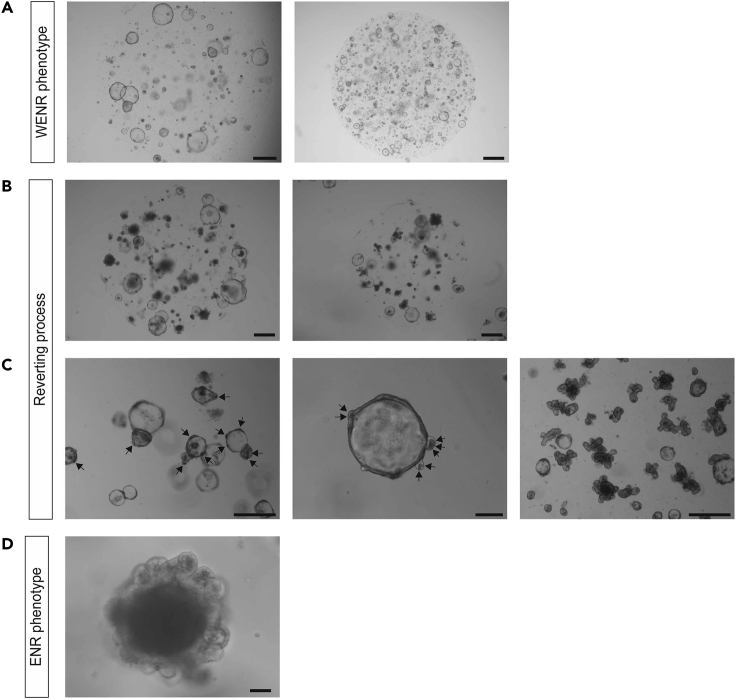
Mouse Small Intestinal Organoids Reverting from a Cyst-like State to a Crypt-Villus Architecture (A) Cyst-like organoid structures growing at two different densities in WENR medium. Scale bars, 500 μm. (B-C) Various examples of organoid cultures during the reversion process toward 'budding organoids'. (B) Upon downscaling of WNT (50% WENR + 50% ENR medium), organoids start to shed cells. Some organoids may already revert to crypt morphology. Scale bars, 500 μm. (C) Re-appearance of Paneth cells (arrows) in small buds indicates that the organoids can be passaged on ENR medium. Right: after first passage most organoids reverted back to ENR state. Scale bars: left/right 250 μm and middle 50 μm. (D) Mature organoid with crypt-villus phenotype. Scale bar, 50 μm.

***Note:*** When infecting mouse SI organoids, keep organoids on WENR medium during the selection process. Passaging mouse WENR organoids can be performed by mechanical fragmentation using stacked pipet tips (e.g., stack a p200 tip on top of a p1000 tip and vigorously pipet up and down). Subsequently, organoids can be reverted to a crypt-like state in a 2-step process: First, culture organoids for 2–7 days in medium consisting of 50% WENR and 50% ENR. As soon as Paneth cells have reformed (recognizable by their dark and granular appearance inside small budding structures on a bright field table-top microscope), the organoids can be maintained in ENR medium. As not all organoids revert at the same speed, it is advisable to carefully monitor the culture daily and base the timing of media change on the average organoid morphology. Different stages of reverting organoids are depicted in Figure 5.

Troubleshoot 5: STAR signal in resistant organoids.

### Establishing a Clonal STAR Organoid Line

The outgrowth of isolated single cells into organoids is a highly inefficient process. Therefore, organoids need to be grown at a low-to-intermediate density prior to picking to ensure outgrowth while maintaining clonality. (A plating density of approximately 2,000–10,000 cells/100 μL Matrigel is recommended.)

**CRITICAL:** The organoid density should be monitored carefully to ensure that no organoids are fusing during the outgrowth process. For organoid lines which are very sensitive to density, this process might involve plating cells at a density supporting single cell outgrowth and over the course of the next weeks replating intact organoid structures (without disruption) into larger volumes to ensure sufficient space for clonal organoid growth.

**CRITICAL:** The organoid should exceed a size of 300 cells prior to picking, to ensure successful passaging (approx. size depicted in Figure 4E, right panel). Growing the organoids as big as possible while ensuring their clonality and suitable culturing conditions will increase the success rate in establishing clonal organoid lines.

There are three time points at which a clonal organoid can be picked (ordered according to our preference):

• after selection on the initial plating

***Note:*** This option does not require dissociation to single cells. Therefore, it is very suitable for sensitive lines and can also be applied to mouse SI organoids which are growing in their regular crypt-like budding structure (ENR medium).

• after selection and subsequent FACS purification of single STAR^+^ cells

***Note:*** Broadly applicable strategy given that STAR^+^ cells constitute the stem cell population, i.e., can regenerate the organoid culture.

• after selection and subsequent thorough trypsinization to single cells.

***Note:*** This option is preferable in organoid systems in which STAR^+^ cells do not constitute the (sole) stem cell population. For instance, some lung and stomach cancers show upregulation of ASCL2, while it is not expressed during homeostasis (Hu et al., 2016; Huang et al., 2018; Zuo et al., 2018). Thus, the nature of STAR^+^ cells in respective cancer organoids is not well defined yet.

***Note:*** Before you start: Label Eppendorf collection tubes and put them on ice

***Optional:*** Add 10 μL of Advanced DMEM/F12 into each collection tube to more easily release the organoids into medium.

**Timing: 1 h (may be longer if multiple clones are picked)**

100. Select a Matrigel droplet with a big STAR fluorescent organoid using a table-top microscope.
101. Discard culture medium of the selected culture well.
102. Use 50 μL ice-cold (4°C) Advanced DMEM/F12 to dissolve selected Matrigel droplet by tilting the culture plate and placing a pipette-tip (200 μL) above the droplet.
103. Take up the Matrigel droplet and transfer it into a 15 mL falcon tube.
104. ***Optional:*** Add 200 μL 1× dispase to the tube and incubate at 37°C for 5 min until Matrigel is properly dissolved.
105. Top up to 10 mL with ice-cold Advanced DMEM/F12 and carefully pellet organoids by spinning at 300 × *g* at 4°C for 4 min.
106. Take off medium and carefully resuspend organoids in 100 μL Advanced DMEM/F12.

***Note:*** If there are many organoids, increase the volume of Advanced DMEM/F12 to facilitate the next steps.

**Figure 6:**
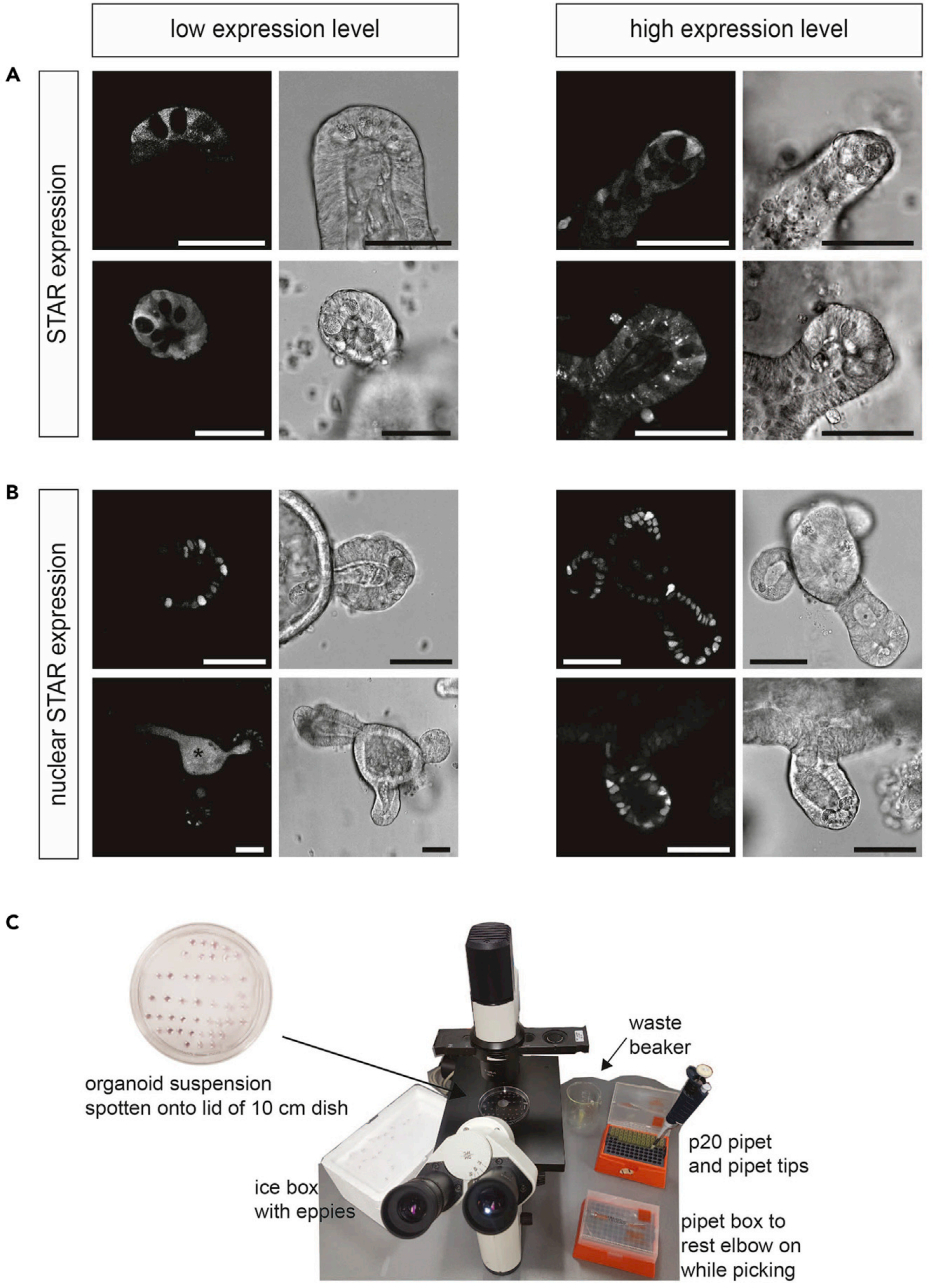
Selecting and Picking a Clonal STAR Organoid (A and B) Organoids with low and high STAR expression levels (white) are depicted on the left and right, respectively. (A) Four examples of crypt structures with STAR expression (uniform cell labeling) that demonstrate clear separation between stem cell niche and TA-region and exclusion of Paneth cells. (B) Four examples of clonal organoids with nuclear STAR expression. (∗) auto-fluorescence. Scale bars, 50 μm. (C) Example setup for picking of clonal organoids using a benchtop microscope.
108. Locate organoids using a table-top or stereomicroscope.
109. For droplets having just one organoid, take up the medium droplet and transfer the organoid into a separate collection tube by carefully pipetting up and down. Verify that the organoid is transferred. If multiple organoids are in the same droplet, carefully take up single organoids using a 20 μL pipet, while checking through the oculars that the other organoids stay put. Transfer organoid into an Eppendorf tube (Figure 6C).
110. Keep Eppendorf tubes on ice while picking.
111. Add 100 μL fresh Trypsin EDTA and incubate at 37°C for 5 min, while vortexing every minute.

***Note:*** This step is recommended for most organoid lines as it allows for the highest plating density. Mouse SI organoids, however, and organoids with a low single cell plating efficiency may benefit from mechanical disruption instead.

107. Spot organoid suspension onto the inside of the lid of a 10 cm culture dish, while making droplets of approximately 20 μL volume (Figure 6C).

***Alternatives:* (to step 114)** Mechanically disrupt organoid by adding 200 μL cold Advanced DMEM and pipetting up and down with a p200 tip several times.

112. Check under the microscope that the organoids has dissociated into small fragments/single cells. Otherwise, prolong the trypsin incubation step.
113. Add 100 μL trypsin inhibitor and top up to 1 mL with Advanced DMEM.
114. Spin Eppendorf tube at 4°C, 500 × *g* for 5 min.
115. Carefully take of supernatant, while leaving 5–10 μL supernatant to not agitate the pellet

Troubleshoot 6: Cell loss when picking clonal organoids

116. Add 30–50 μL Matrigel and mix gently by pipetting up and down.
117. Plate Matrigel in 2 droplets onto a pre-warmed 48-well plate with one droplet per well.

***Note:*** Efficient clonal outgrowth requires at least 5 crypt-like structures (mouse small intestinal organoids in ENR state) or 100 single cells per Matrigel droplet of 10 μL.

118. Incubate the plate upside-down in the incubator for 10–25 min until the Matrigel has solidified
119. Add 250 μL organoid medium with 10 μM Y-27632 and 100 μg/mL primocin (since clonal organoids has been exposed to an environment outside the hood).

***Note:*** In case of a low yield or organoid lines that are particularly sensitive to low density, it may be conceivable to plate STAR^+^ cells together with reporter-negative cells to support organoid growth. FACS purification can be subsequently used to isolate all STAR^+^ cells. Troubleshoot 7: Cell death after passaging clonal organoids

## Expected Outcomes

STAR activity reflects the transcriptional activity of the intestinal stem cell specific transcription factor ASCL2. Hence, STAR labels intestinal stem cells and, with decreasing intensity along the crypt-villus axis, transit amplifying cells (but not differentiated cells). The overall intensity levels depend on the number of ASCL2-binding motif repeats (STAR repeats, compare Figure 2), the brightness of the fluorophore, and the laser intensity used for exposure. While the nature of the STAR intensity gradient along the crypt-villus axis is independent of these parameters, the absolute intensity impacts on the visual manifestation of the gradient (Figures 6A and 6B). We therefore recommend analyzing multiple clonal organoids and compare their STAR expression to get the best fit for your research question.

1. Plate clonal STAR organoid cultures on glass for live-cell microscopy.

***Note:*** It is convenient to have multiple clonal lines (> 3 clonal lines) to compare them with each other. In the end the aim is to report both reliable and efficient ASCL2 activity in stem cells.

2. Image STAR expression levels and pick your favorite. The range of STAR pattering in clonal organoids is depicted in Figures 6A and 6B.

## Limitations

STAR reports the transcriptional activity of ASCL2, thus its application is limited to tissues that express ASCL2 (such as the small and large intestine, placenta, and several tumor tissues) (Jubb et al., 2006, The Human Protein Atlas). The recent discovery of ASCL2 controlling the LGR5^+^ stem cell state in the intestine, makes STAR a versatile tool to report intestinal stemness which partially overlaps but is more exclusive than Wnt activity (Murata et al., 2020; Oost et al., 2018; Schuijers et al., 2015). While STAR has been successfully applied in mouse and human intestinal tissues, extending the range to other species and tissues is hitherto untested. Furthermore, for tissues in which ASCL2 is expressed by a differentiated cell type rather than the stem cell compartment, reporter plasmids with STAR-driven expression of the antibiotic selection cassette are not suitable. It is advisable therefore to choose reporter plasmids with an independent, uniform promoter which drives the expression of a selection cassette and works in parallel to STAR (see Table 1).

When analyzing cell-type conversions, it is important to note that while the STAR signal is maintained by ongoing ASCL2 transcriptional activity, its decay is mainly determined by the half-life of the fluorescent protein itself and not directly related to the stability of ASCL2. Additionally, expansion media for organoids are generally rich in stem cell supporting factors which, in turn, can lead to widespread STAR activity at varying levels within a single organoid. For instance, with respect to the intestine, this reflects the stemness gradient from the crypt bottoms up to the transit amplifying zone. Please note, conditions that favor cellular differentiation, either by modulating the media composition or xenotransplantation in mice which provides a more native micro-environment, facilitates cellular diversity and might be desirable depending on the organoid system and the research question (Fumagalli et al., 2020; Oost et al., 2018).

## Troubleshooting

### Problem 1

STAR will not integrate into the genome.

### Potential Solution for Lentiviral Approach (Protocol A)

Infecting organoids requires a much higher virus titre than most cell lines. Therefore, concentration of the virus prior to transduction and the addition of polybrene may be key to success. To test for successful virus production, a cell line of choice can be transduced alongside with the organoids (using a small split of the virus suspension). The STAR signal should be visible after 1–2 days. If the cell line does not express ASCL2, transient transfection of ASCL2 (and oncogenic beta-catenin) is required to test for successful transduction (see Troubleshoot 8: How to functionally test the STAR plasmid”). Note, however, that while this test can be easily performed alongside with the actual transduction, it is inconclusive on the amount of virus particles being produced.

### Potential Solution for Transposon-Based Approach with Electroporation (Protocol B)

Double (or triple) the amount of STAR reporter DNA and of Tol2 transposase. When viability is not the limiting step, you may increase the poring pulse (up to 220 Ω in steps of 10 Ω for instance) and/or poring pulse length (up to10 ms, in steps of 1 ms).

### Potential Solution for Transposon-Based Approach with Transfection Reagent (Protocol C)

If transfection with a transfection reagent does not work well, consider using the transfection with electroporation protocol (protocol B).

### Problem 2

No access to the indicated lentiviral envelope, packaging vectors, or transfection reagent.

### Potential Solution

The three plasmids pHDM-G, pHDM-Hgpm2, and pRC-CMV-rev1b lead to the expression of VSV-G, gag and pol, and rev, respectively. Alternative plasmids containing these functional elements are commercially available, for instance on Addgene.

Alternatively to X-tremeGENE 9, the authors have successfully used TransIT-LT1 (Sopachem, MIR 2360) for the transfection of 293T cells during virus making (protocol A1, with a ratio of 2 μL transfection reagent for 1 μg of DNA). However, the transfection of STAR into organoids using a transfection reagent (protocol C2) has so far only been performed with X-tremeGENE 9 by the authors. While other transfection reagents might be suitable as well, we cannot comment on necessary adaptations to the existing protocol.

### Problem 3

The cells die upon transduction with STAR lentiviruses.

### Potential Solution

The virus titre might be too high. We suggest to set up a small dilution range of the virus (following ultracentrifugation) and transduce cells with different virus titres. If the cells are readily transducable, like some cell lines, ultracentrifugation is obsolete as the viral supernatant can be directly harvested of the virus-producing 293T cells, filtered with a 45 μm strainer (see protocol A step 9), supplemented with 8 μg/mL polybrene, and added on top of the receiving cells.

### Problem 4

Electroporation kills all cells.

### Potential Solution

Use up to 1 million cells per electroporation (while ensuring that the impedance is within the limits of 30–55 Ω). If still unsuccessful, lower the poring voltage and/or poring pulse length to make the procedure less harsh.

### Problem 5

The cells are resistant to the selection antibiotic but there is no STAR signal.

### Potential Solution

For plasmids in which the antibiotics selection cassette is controlled by an independent promoter, it is conceivable that expression of the reporter gene is compromised by epigenetic silencing of the STAR promoter/enhancer site, while the ubiquitous promoter is still active. If this applies to a subculture, FACS purification of STAR^+^ cells or establishing a clonal line from STAR^+^ cells may solve the problem. Otherwise, when working with colorectal cancer organoids, make sure ASCL2 is still expressed in this tissue. You may also consider to integrate insulator sequences around the reporter to reduce the risk of epigenetic silencing (Ramezani et al., 2003). Note, however, that insulator sequences might be detrimental for the virus titre (Hanawa et al., 2009).

### Problem 6

Loss of cells while picking clonal organoids.

### Potential Solution

Pre-coat Eppendorf tubes with 10% fetal bovine serum or 1% BSA to prevent the cells from sticking to the plastic. If the cells easily stick to the pipet tip (check under the microscope), consider to pre-coat the tips as well. After trypsinizing the organoid and spinning down the single cell suspension, check under a table-top microscope to identify the location of the pellet. Carefully tilt the Eppendorf tube and take off media in multiple steps while checking that the pellet stays intact after each pipetting step. Collect supernatant in second Eppendorf tube to make sure the cells can be recovered in case the pellet is loose. Alternatively use 15 mL falcon tubes to get a cleaner pellet.

### Problem 7

When generating clonal lines, the cells derived from a single organoid have difficulties surviving after the first passage.

### Potential Solution

Organoid cells require a minimum density for successful outgrowth. Consider plating the cells in the presence of STAR-negative supporter cells of the same line to increase the plating density. This steps requires subsequent purification of STAR^+^ cells at a later time-point (e.g., by FACS).

### Problem 8

How to functionally test the STAR plasmid.

### Potential Solution

Transiently transfect a cell line of your choice with the STAR plasmid. If the cells express ASCL2, the fluorescent protein should be visible under a confocal laser microscope. As 293T cells do not express ASCL2, STAR plasmids can be tested by co-transfecting ASCL2 (and oncogenic beta-catenin) to stimulate ASCL2 activity. For suitable plasmids, see Schuijers et al (Schuijers et al., 2015).

### Problem 9

No access to growth factor conditioned media.

### Potential Solution

Wnt 3A, Noggin, and R-Spondin 1 conditioned media can be replaced by the Wnt Surrogate-Fc fusion protein, the Noggin-FC fusion protein conditioned medium, and the R-Spondin 3-FC fusion protein conditioned medium, respectively (U-Protein Express, Cat. No. #N001, #N002, and #R001, respectively).

The recommended concentration of the Wnt Surrogate-Fc fusion protein (U-protein Express, Cat.No. #N001) is around 0.1–0.5 μM. It should be noted, however, that the optimal concentration might vary per organoid line. The recombinant Noggin and R-Spondin 3 may be used 1:100.

Other recombinant proteins are commercially available and might be suitable as well but the authors do not have any experience with them.

## Resource Availability

### Lead Contact

Further information and requests for resources and reagents should be directed to and will be fulfilled by the Lead Contact, Dr Hugo Snippert (H.J.G.Snippert@umcutrecht.nl).

### Materials Availability

STAR plasmids referred to this study have been deposited to Addgene as listed in the Table 1. Additional plasmids required to perform the protocols are commercially available (Addgene or plasmID) and are listed in the Key Resources Table.

### Data and Code Availability

The sequences of the STAR plasmids and the Tol2 transposase are available on Addgene via the Addgene numbers indicated in Table 1 and the Key Resources Table.

## References

[bib1] Andersson-Rolf A., Fink J., Mustata R.C., Koo B.K. (2014). A video protocol of retroviral infection in primary intestinal Organoid culture. J. Vis. Exp..

[bib2] Drost J., van Jaarsveld R.H., Ponsioen B., Zimberlin C., van Boxtel R., Buijs A., Sachs N., Overmeer R.M., Offerhaus G.J., Begthel H. (2015). Sequential cancer mutations in cultured human intestinal stem cells. Nature.

[bib3] Van der Flier L.G., Sabates-Bellver J., Oving I., Haegebarth A., De Palo M., Anti M., Van Gijn M.E., Suijkerbuijk S., Van de Wetering M., Marra G. (2007). The Intestinal Wnt/TCF Signature. Gastroenterology.

[bib4] Fujii M., Matano M., Nanki K., Sato T. (2015). Efficient genetic engineering of human intestinal organoids using electroporation. Nat. Protoc..

[bib5] Fujii M., Matano M., Toshimitsu K., Takano A., Mikami Y., Nishikori S., Sugimoto S., Sato T. (2018). Human intestinal organoids maintain self-renewal capacity and cellular diversity in niche-inspired culture condition. Cell Stem Cell.

[bib6] Fumagalli A., Oost K.C., Kester L., Morgner J., Bornes L., Bruens L., Spaargaren L., Azkanaz M., Schelfhorst T., Beerling E. (2020). Plasticity of Lgr5-negative cancer cells drives metastasis in colorectal cancer. Cell Stem Cell.

[bib7] Hanawa H., Yamamoto M., Zhao H., Shimada T., Persons D.A. (2009). Optimized lentiviral vector design improves titer and transgene expression of vectors containing the chicken β-globin locus HS4 insulator element. Mol. Ther..

[bib8] Hu X., Chen L., Wang Q., Zhao X., Tan J., Cui Y., Liu X., Zhang X., Bian X. (2016). Elevated expression of ASCL2 is an independent prognostic indicator in lung squamous cell carcinoma. J. Clin. Pathol..

[bib9] Huang Y.H., Klingbeil O., He X.Y., Wu X.S., Arun G., Lu B., Somerville T.D.D., Milazzo J.P., Wilkinson J.E., Demerdash O.E. (2018). POU2F3 is a master regulator of a tuft cell-like variant of small cell lung cancer. Genes Dev..

[bib10] Jubb A., Chalasani S., Frantz G., Smits R., Grabsch H., Kavi V., Maughan N., Hillan K., Quirke P., Koeppen H. (2006). Achaete-scute like 2 (ascl2) is a target of Wnt signalling and is upregulated in intestinal neoplasia. Oncogene.

[bib11] Koo B.K., Stange D.E., Sato T., Karthaus W., Farin H.F., Huch M., Van Es J.H., Clevers H. (2012). Controlled gene expression in primary Lgr5 organoid cultures. Nat. Methods.

[bib12] Van Lidth de Jeude J.F., Vermeulen J.L.M., Montenegro-Miranda P.S., Van den Brink G.R., Heijmans J. (2015). A protocol for lentiviral transduction and downstream analysis of intestinal organoids. J. Vis. Exp..

[bib13] Murata K., Jadhav U., Madha S., van Es J., Dean J., Cavazza A., Wucherpfennig K., Michor F., Clevers H., Shivdasani R.A. (2020). Ascl2-Dependent Cell Dedifferentiation Drives Regeneration of Ablated Intestinal Stem Cells. Cell Stem Cell.

[bib14] Oost K.C., van Voorthuijsen L., Fumagalli A., Lindeboom R.G.H., Sprangers J., Omerzu M., Rodriguez-Colman M.J., Heinz M.C., Verlaan-Klink I., Maurice M.M. (2018). Specific Labeling of Stem Cell Activity in Human Colorectal Organoids Using an ASCL2-Responsive Minigene. Cell Rep..

[bib15] Ramezani A., Hawley T.S., Hawley R.G. (2003). Performance- and safety-enhanced lentiviral vectors containing the human interferon-β scaffold attachment region and the chicken β-globin insulator. Blood.

[bib16] Sato T., Vries R.G., Snippert H.J., van de Wetering M., Barker N., Stange D.E., van Es J.H., Abo A., Kujala P., Peters P.J. (2009). Single Lgr5 stem cells build crypt-villus structures in vitro without a mesenchymal niche. Nature.

[bib17] Sato T., Stange D.E., Ferrante M., Vries R.G.J., van Es J.H., van Den Brink S., van Houdt W.J., Pronk A., van Gorp J., Siersema P.D. (2011). Long-term expansion of epithelial organoids from human colon, adenoma, adenocarcinoma, and Barrett’s epithelium. Gastroenterology.

[bib18] Schuijers J., Junker J.P., Mokry M., Hatzis P., Koo B.K., Sasselli V., Van Der Flier L.G., Cuppen E., Van Oudenaarden A., Clevers H. (2015). Ascl2 acts as an R-spondin/Wnt-responsive switch to control stemness in intestinal crypts. Cell Stem Cell.

[bib19] Schwank G., Andersson-Rolf A., Koo B.-K., Sasaki N., Clevers H. (2013). Generation of BAC Transgenic Epithelial Organoids. PLoS One.

[bib20] Shimokawa M., Ohta Y., Nishikori S., Matano M., Takano A., Fujii M., Date S., Sugimoto S., Kanai T., Sato T. (2017). Visualization and targeting of LGR5+ human colon cancer stem cells. Nature.

[bib21] Sugimoto S., Ohta Y., Fujii M., Matano M., Shimokawa M., Nanki K., Date S., Nishikori S., Nakazato Y., Nakamura T. (2017). Reconstruction of the human colon epithelium in vivo. Cell Stem Cell.

[bib24] (2020). https://www.proteinatlas.org/ENSG00000183734-ASCL2/tissue.

[bib22] Ziskin J.L., Dunlap D., Yaylaoglu M., Fodor I.K., Forrest W.F., Patel R., Ge N., Hutchins G.G., Pine J.K., Quirke P. (2013). In situ validation of an intestinal stem cell signature in colorectal cancer. Gut.

[bib23] Zuo Q., Wang J., Chen C., Zhang Y., Feng D.-X., Zhao R., Chen T. (2018). ASCL2 expression contributes to gastric tumor migration and invasion by downregulating miR223 and inducing EMT. Mol. Med. Rep..

